# MeJA inhibits fungal growth and DON toxin production by interfering with the cAMP-PKA signaling pathway in the wheat scab fungus *Fusarium graminearum*

**DOI:** 10.1128/mbio.03151-24

**Published:** 2025-02-04

**Authors:** Kaili Duan, Shaozhe Qin, Fangling Cui, Liangyuan Zhao, Yongqing Huang, Jin-Rong Xu, Guanghui Wang

**Affiliations:** 1 State Key Laboratory for Crop Stress Resistance and High-Efficiency Production, College of Plant Protection, Northwest A&F University, Yangling, Shaanxi, China; 2 Department of Botany and Plant Pathology, Purdue University, West Lafayette, Indiana, USA; Universidade de Sao Paulo, Ribeirao Preto, Sao Paulo, Brazil

**Keywords:** methyl jasmonate, deoxynivalenol, PKA signaling pathway, MAP kinase pathway, *Fusarium graminearum*

## Abstract

Deoxynivalenol (DON), a mycotoxin primarily produced by *Fusarium* species, is commonly found in cereal grains and poses risks to human and animal health, as well as global grain trade. This study demonstrates that methyl jasmonate (MeJA), a natural plant hormone, inhibits the growth and conidiation of *Fusarium graminearum*. Importantly, MeJA significantly reduces DON production by suppressing *TRI* gene expression and toxisome formation. To explore the molecular mechanism, we identified MeJA-tolerant mutants, including a transcription factor *MRT1* and cAMP-PKA pathway-related genes (*FgGPA1* and *FgSNT1*). MeJA treatment reduced PKA activity and intracellular cAMP levels in *F. graminearum*, suggesting it targets the cAMP-PKA pathway. Notably, the MeJA-resistant mutant *FgGPA1*^R178H^ enhanced fungal growth, DON production, and cAMP levels in the presence of MeJA. Exogenous cAMP alleviated MeJA’s inhibitory effects on DON production, further supporting this pathway’s involvement. Interestingly, MeJA had no effect on all three MAP kinase pathways (Mgv1, Gpmk1, and FgHog1). Truncated and phospho-mimicking mutations in Mrt1 or FgSnt1 conferred MeJA resistance, suggesting they may act downstream of the cAMP-PKA pathway. In conclusion, MeJA presents a promising approach to control *F. graminearum* growth and DON production.

**IMPORTANCE:**

Deoxynivalenol (DON) poses significant risks to both human and animal health and severely disrupts the global grain trade due to its prevalence as a common contaminant in wheat grains. With rising public concern over food safety, finding effective and sustainable methods to reduce DON contamination becomes increasingly urgent. In our study, we found that methyl jasmonate (MeJA), a natural plant hormone, can effectively inhibit the vegetative growth of *F. graminearum* and significantly reduce its DON toxin production. To explore the underlying molecular mechanism, we identified the mutations in MeJA-tolerant mutants and revealed that MeJA effectively exerts its antifungal activities by inhibiting the cAMP-PKA signaling pathway in *F. graminearum*. Our work provides a promising natural solution to reduce DON toxin contamination in cereal grains, enhancing food safety while decreasing the reliance on chemical fungicides and their associated environmental impact.

## INTRODUCTION

Deoxynivalenol (DON), a type B trichothecene mycotoxin produced by *Fusarium* species, is the most prevalent contaminant in cereals and cereal-derived products (1, 2). The DON toxin exerts severely detrimental effects on a range of vital cellular processes in eukaryotes, including protein synthesis inhibition, membrane structure damage, and mitochondrial dysfunction (3). When ingested by humans or animals, it triggers a spectrum of acute and chronic toxic effects, such as anorexia, vomiting, growth failure, and reproductive dysfunction (4, 5). Moreover, it is considered carcinogenic by the World Health Organization (6). *Fusarium* head blight (FHB), a highly devastating disease of wheat worldwide, is caused by a *Fusarium* species complex, with *Fusarium graminearum* as the primary causal agent (7, 8). This fungus initially infects the wheat spike during flower opening, with high humidity and warm temperatures facilitating the infection (9). FHB is characterized by blighted, shriveled wheat heads and lightweight kernels, resulting in yield losses of up to 80% (10). Even worse, FHB contaminates the infected grain with harmful DON toxin, posing a serious threat to food safety (1). DON is also a virulence factor promoting *F. graminearum* to cross the rachis node during wheat head infection (11). Since the 1970s, the FHB-affected regions have expanded rapidly and are predicted to increase with a changing climate, seriously threatening global wheat production (12). In addition to yield losses, the food safety issues caused by DON toxin have aroused growing public concern. Therefore, it is absolutely urgent to protect crops from DON contamination to ensure food safety.

Trichothecenes are biosynthesized from farnesyl pyrophosphate (FPP), which is produced from acetyl-CoA through the mevalonate pathway (13). *TRI5* encodes an enzyme that catalyzes the first step of this synthesis, specifically converting FPP into trichodiene (11). *TRI5* is located in the core *TRI* gene cluster on chromosome 2, which includes 12 *TRI* genes (14), whereas three remaining *TRI* genes are located at a two-gene locus (*TRI1* and *TRI16*) and a single-gene locus (*TRI101*) (15, 16). Within the core *TRI* gene cluster, seven genes (*TRI3*, *TRI4*, *TRI5*, *TRI7*, *TRI8*, *TRI11*, and *TRI13*) encode enzymes. Additionally, *TRI6* and *TRI10* encode two key transcriptional regulators for *TRI* gene expression (17, 18), while *TRI12* encodes a major facilitator superfamily transporter involved in DON efflux (19). Various environmental and nutritional factors, including nitrogen sources, carbon sources, pH, and reactive oxygen species (ROS), facilitate DON production in *F. graminearum* (20–24). However, how these environmental factors affect DON biosynthesis is largely unknown.

The cAMP-PKA signaling pathway, a major signal transduction pathway in fungi, plays important roles in fungal development, secondary metabolism, and pathogenesis (25, 26). In the absence of cAMP, the PKA holoenzyme exists in an inactive form as a heterotetramer composed of two regulatory (R) and two catalytic (C) subunits. However, upon exposure to cAMP, the binding of cAMP to the R subunits triggers the activation of two monomeric C subunits (27). This activation results in the phosphorylation of downstream targets, ultimately regulating a diverse array of cellular functions (28). In *F. graminearum*, several key components of the cAMP-PKA pathway are involved in DON toxin synthesis, including catalytic subunits (Cpk1 and Cpk2), regulatory subunit (Pkr), adenylate cyclase (Fac1), two cAMP phosphodiesterases (Pde1 and Pde2), and an adenylate cyclase-associated protein (FgCap1) (29). Furthermore, a total of three MAP kinases had been identified in *F. graminearum*, including Gpmk1, Mgv1, and FgHog1 MAP kinases that also play critical roles in DON production (30, 31). However, how cAMP-PKA and MAPK signaling pathways regulate DON biosynthesis is unclear.

Jasmonic acid (JA) and its methylated derivative methyl jasmonate (MeJA) are important phytohormones in regulating plant resistance to biotic and abiotic stresses, including pathogen attacks and environmental fluctuations (32, 33). JA is well documented for its function in orchestrating plant defense responses, whereas MeJA has been extensively studied for its potential to induce robust defense responses in plants (32, 34–36). Although several studies have reported that MeJA exhibits direct antifungal activities against some fungal pathogens, the underlying mechanism is unclear (37–39).

In this study, we demonstrate that MeJA not only inhibits fungal growth and conidiation in *F. graminearum* but also significantly reduces DON production. Through whole-genome sequencing, we identified mutations conferring MeJA resistance in several genes, including a transcriptional factor *MRT1* and two cAMP-PKA pathway-related genes (*FgGPA1* and *FgSNT1*). We further provided evidence that MeJA exerts its antifungal activities by inhibiting the cAMP-PKA signaling pathway in *F. graminearum*, while Mrt1 and FgSnt1 function downstream of the PKA pathway and confer MeJA resistance likely by altering gene transcription.

## RESULTS

### MeJA significantly inhibits the vegetative growth and conidiation in *F. graminearum*

**Fig 1:**
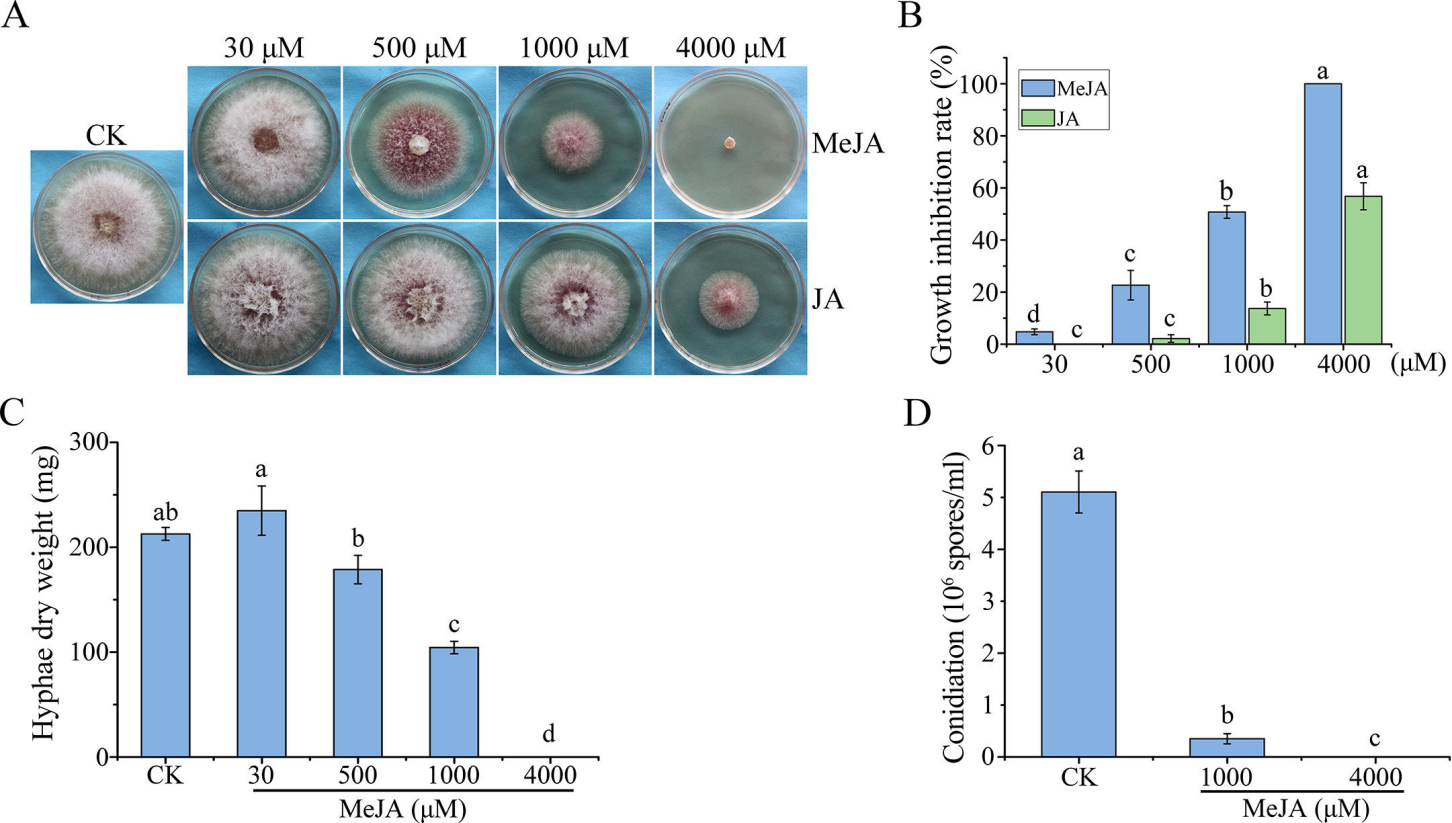
Assays for the inhibitory effects of MeJA and JA on hyphal growth and conidiation. (A) Three-day-old PDA cultures of PH-1 strain with different concentrations (30, 500, 1,000, and 4,000 µM) of MeJA or JA. (B) The growth inhibition rates of the PH-1 strain treated with indicated concentrations of MeJA or JA on PDA plates. (C) Mycelium of PH-1, harvested from 24-h YEPD cultures containing 30, 500, 1,000, and 4,000 µM MeJA, was lyophilized and used to measure the dry weight. (D) Conidiation of PH-1 in 5-day-old CMC cultures with 0.1% ethanol (CK) and 1,000 or 4,000 µM MeJA. Mean and standard deviation were estimated with data from three independent biological replicates. Different letters indicate significant differences with the 0.1% ethanol control (CK) based on ANOVA followed by Tukey’s range tests (*P* < 0.05).

To determine the effect of jasmonates on hyphal growth, we treated the wild-type strain PH-1 with different concentrations of MeJA and JA. On potato dextrose agar (PDA) plates, both of them had dosage-dependent inhibitory effects on hyphal growth (Fig. 1A). Under the same concentrations, MeJA was more effective in inhibiting *F. graminearum* growth than JA. At 4,000 µM, MeJA almost completely inhibited colony growth but JA only reduced growth rate by 56.7% (Fig. 1B). At 500 µM, MeJA significantly reduced colony growth but JA had no obvious effect on *F. graminearum* (Fig. 1A). We then assayed the inhibitory effect of MeJA on *F. graminearum* by measuring the mycelial biomass in liquid yeast extract peptone dextrose (YEPD) medium. Treatments with 500 and 1,000 µM MeJA reduced the dry biomasses of PH-1 by 16.0% and 50.9%, respectively (Fig. 1C). Hyphal biomass was barely detectable when treated with 4,000 µM MeJA (Fig. 1C). These results confirmed that MeJA has a dosage-dependent inhibitory effect on vegetative growth in *F. graminearum*.

We also investigated the effects of MeJA on conidial production in *F. graminearum*. Treatments with 1,000 µM MeJA significantly reduced conidiation (81.9% reduction) compared to the untreated control in 5-day-old CMC cultures (Fig. 1D). When the concentration of MeJA was increased to 4,000 µM, no conidia were observed (Fig. 1D). These results indicated that MeJA significantly inhibits conidiation in *F. graminearum*.

### MeJA significantly reduces DON production, *TRI* gene expression, and toxisome formation

**Fig 2:**
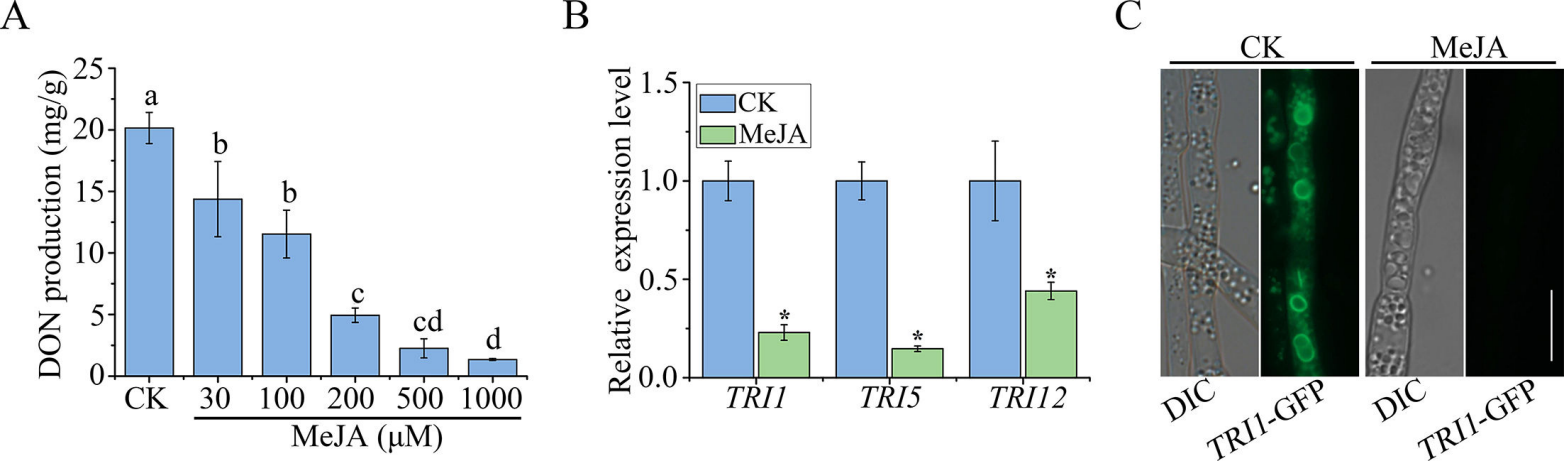
Effects of MeJA on DON production and *TRI* gene expression. (A) DON production of PH-1 in LTB cultures with different concentrations (30, 100, 200, 500, and 1,000 µM) of MeJA or 0.1% ethanol (CK) at 7 days post-inoculation. Different letters indicate significant differences based on ANOVA followed by Tukey’s range tests (*P* < 0.05). (B) The expression level of *TRI1*, *TRI5*, and *TRI12* was assayed by qRT-PCR with RNA isolated from hyphae in 3-day-old LTB cultures with 1,000 µM MeJA. The relative *TRI* gene expression level in each CK was set to 1. Asterisks indicate significant differences in comparison with CK based on ANOVA (*P* < 0.05). Mean and standard deviation were calculated with data from three independent replicates. (C) Three-day-old LTB cultures of *TRI1-GFP* transformant treated with or without 1,000 µM MeJA were examined for the expression and localization of Tri1-GFP. Bar = 10 µm.

Since DON is the most prevalent harmful toxin in wheat grains, we also investigated whether MeJA has an inhibitory effect on DON production. Treatments with 100 and 200 µM MeJA resulted in a 42.8% and 75.5% reduction in DON production, respectively, when compared to the control in LTB (liquid trichothecene biosynthesis) cultures (Fig. 2A). At 1,000 µM, MeJA almost completely blocked DON production (Fig. 2A). When assayed by qRT-PCR, the expression levels of *TRI1*, *TRI5*, and *TRI12* genes were reduced more than 50% in samples treated with 1,000 µM MeJA (Fig. 2B).

Toxisomes are special spherical structures formed by *F. graminearum* during DON toxin biosynthesis, which are presumed to be the sites for DON biosynthesis (40). Both Tri1 and Tri4, two cytochrome P-450 oxygenases, accumulate in the periphery of toxisomes and are frequently used as markers for toxisome observation (40). To investigate the effect of MeJA on toxisome formation in *F. graminearum*, we generated a transformant of PH-1 expressing the *TRI1*-GFP construct (Table 1). In untreated samples, abundant toxisomes were observed. However, no toxisome was observed in samples treated with 1,000 µM MeJA (Fig. 2C), indicating that MeJA inhibits the formation of toxisomes. Taken together, our results indicate that MeJA is inhibitory to DON production, likely by inhibiting *TRI* gene expression and toxisome formation.

**TABLE 1:** Wild-type and mutant strains of *F. graminearum* used in this study

Strain	Description	Source
WT	Wild-type strain PH-1	(41)
*TRI1*-GFP	PH-1 expressing *TRI1*-GFP	(42)
*mrt1*	*mrt1* deletion mutant of PH-1	This study
*Fggpa1*	*Fggpa1* deletion mutant of PH-1	This study
*pde2*	*pde2* deletion mutant of PH-1	(17)
*MRT1*^∆CT171^-1	Transformant of *mrt1* mutant expressing *MRT1*^∆CT171^ construct	This study
*MRT1*^∆CT171^-2	Transformant of *mrt1* mutant expressing *MRT1*^∆CT171^ construct	This study
*FgSNT1*^∆CT179^-2	*FgSNT1*^∆CT179^ deletion mutant of PH-1	This study
*FgSNT1*^∆CT179^-4	*FgSNT1*^∆CT179^ deletion mutant of PH-1	This study
*FgGPA1*^R178H^-6	Transformant of *Fggpa1* mutant expressing *FgGPA1*^R178H^ construct	This study
*FgGPA1*^R178H^-10	Transformant of *Fggpa1* mutant expressing *FgGPA1*^R178H^ construct	This study
*FgGPA1*^R178C^-1	Transformant of *Fggpa1* mutant expressing *FgGPA1*^R178C^ construct	This study
*FgGPA1*^R178C^-3	Transformant of *Fggpa1* mutant expressing *FgGPA1*^R178C^ construct	This study
*MRT1* ^S177A^	Transformant of *mrt1* mutant expressing *MRT1*^S177A^ construct	This study
*MRT1* ^S177D^	Transformant of *mrt1* mutant expressing *MRT1*^S177D^ construct	This study
*MRT1* ^T283A^	Transformant of *mrt1* mutant expressing *MRT1*^T283A^ construct	This study
*MRT1*^T283D^-1	Transformant of *mrt1* mutant expressing *MRT1*^T283D^ construct	This study
*MRT1*^T283D^-2	Transformant of *mrt1* mutant expressing *MRT1*^T283D^ construct	This study

We further examined the impact of MeJA on the virulence of *F. graminearum* and its DON production *in planta*. The flowering wheat heads were drop inoculated with conidium suspensions of PH-1 containing 500 or 1,000 µM MeJA. At 14 days post-inoculation (dpi), the MeJA slightly reduced the virulence of *F. graminearum*, though the reduction was not statistically significant (Fig. S1A and B). However, the DON production in the inoculated wheat spikes was significantly reduced (Fig. S1C). We speculated that exogenous MeJA may be degraded by the plant when its concentration exceeds physiological levels, or it may be difficult for MeJA to penetrate the rachis through the dense node.

### MeJA has no effect on intracellular ROS accumulation and cell viability

It has been reported that exogenous MeJA triggers ROS production in *Ganoderma lucidum* (43). To determine whether MeJA elicits ROS production in *F. graminearum*, we assayed ROS accumulation in the germlings of PH-1 treated with 1,000 µM MeJA by staining with 2,7-dichlorodihydrofluorescein diacetate (DCFH-DA), a cell-permeable ROS indicator (44). No intense green fluorescence signals were observed in the cytoplasm of germ tubes, regardless of whether they were treated with 1,000 µM MeJA (Fig. S2A). No significant difference was observed by measuring the fluorescence intensity at excitation and emission wavelengths of 480 and 530 nm, respectively (Fig. S2B). These results indicate that MeJA does not induce the accumulation of intracellular ROS in *F. graminearum*.

We then examined the impact of MeJA on cell viability by staining with propidium iodide (PI), a viability indicator (45). Whereas hyphae subjected to heat treatment at 100°C for 3 min showed strong fluorescent signals, hyphae treated with or without MeJA had no obvious PI staining (Fig. S3). These results indicate that MeJA has no effect on the hyphal cell viability of *F. graminearum*. Thus, other molecular mechanisms should contribute to the inhibitory effects of MeJA on vegetative growth and DON production.

### Identification of mutations in MeJA-resistant mutants

Interestingly, after incubation for extended periods, sectors with faster growth or denser aerial mycelium were occasionally observed in PDA cultures of PH-1 with 4,000 µM MeJA. To uncover the underlying mechanism of MeJA’s inhibitory effects on *F. graminearum*, we isolated a total of 31 MeJA-tolerant spontaneous mutants and verified their resistance to 1,000 µM MeJA (Fig. S4; Table S1).

**Fig 3:**
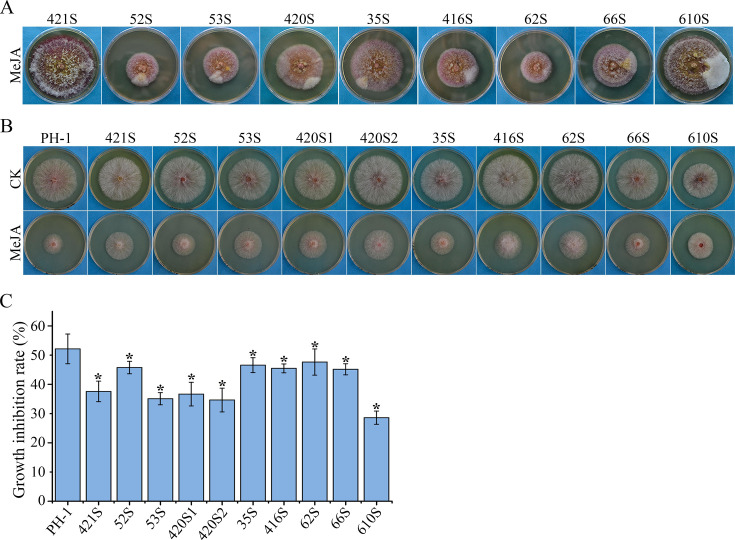
Isolation and examination of the MeJA-resistant mutants. (A) The morphology of PH-1 in PDA cultures with 4,000 µM MeJA after incubation for 10–30 days. (B) The morphology of 10 MeJA-resistant mutants with the relatively lower growth inhibition rates was examined in PDA with or without 1,000 µM MeJA at 3 days post-inoculation. (C) The growth of MeJA-resistant mutants on PDA plates with 1,000 µM MeJA was measured to estimate the growth inhibition rate. Asterisks indicate significant differences in comparison with the PH-1 based on ANOVA (*P* < 0.05).

To determine the genetic basis of MeJA resistance, 10 MeJA-resistant mutants with the lowest growth inhibition rate were selected for whole-genome sequencing analysis (Fig. 3). Mutations were identified in seven genes, including FGSG_03292 (a putative transcription factor), FGSG_00324 (*FgSNT1*, a subunit of Set3C HDAC complex), and FGSG_05535 (*FgGPA1*, the heterotrimeric G-protein Gα subunit) (Table 2). The FGSG_03292 gene, named *MRT1* (for MeJA resistance-associated transcription factor 1) in this study, had four different mutation sites identified in five MeJA-tolerant mutants, including two nonsense mutations (W616* in 420S1 and 53S, W552* in 52S) and two missense mutations (C34F in 66S and A371T in 35S). Five other mutations were detected in *FgSNT1* (Q2056*), *FgGPA1* (R178H), FGSG_12003 (G353C), FGSG_05542 (G1623R), and FGSG_06157 (G249D) (Table 2).

**TABLE 2:** Mutations identified in MeJA-resistant mutants by whole-genome sequencing

MeJA-resistant mutants	Gene ID	Yeast ortholog	Nucleotide changes	Amino acid changes
421S	FGSG_00324	*SNT1*	C6166T	Q2056*^a^
62S	FGSG_05119	YCR023C	G1027A	G343S
66S	FGSG_12003	*DAL4*/*FUR4*	G1057T	G353C
	FGSG_03292	None	G101T	C34F
610S	FGSG_05535	*GPA1*	G533A	R178H
35S	FGSG_03292	None	G1111A	A371T
	FGSG_05542	*NUP188*	G4867A	G1623R
416S	FGSG_05119	YCR023C	G394A	G132S
420S1	FGSG_03292	None	G1848A	W616*
53S	FGSG_03292	None	G1848A	W616*
52S	FGSG_03292	None	G655A	W552*
	FGSG_06157	None	G746A	G249D
420S2	FGSG_05119	YCR023C	G3A	M1I

^*a*^ * represents stop codon.

### The C-terminal region truncation and T283D mutation of *MRT1* increase tolerance against MeJA

**Fig 4:**
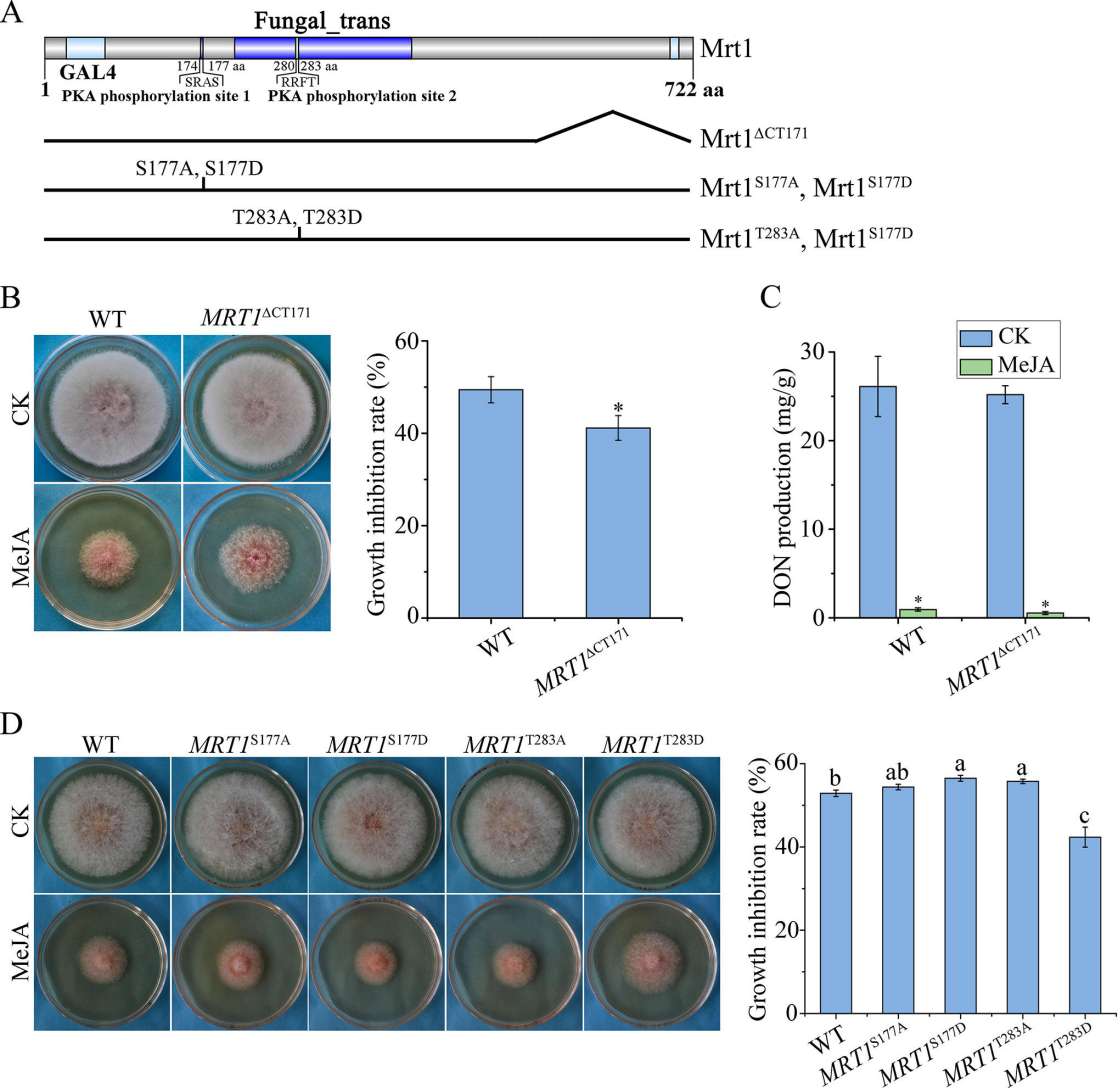
Mutations of *MRT1*^∆CT171^ and *MRT1*^T283D^ increase resistance to MeJA in hyphal growth. (A) Schematic diagram of different mutant alleles of Mrt1. (B) The morphology and growth inhibition rate of wild-type (WT) PH-1 and *MRT1*^∆CT171^ mutant at 3 dpi in PDA plates containing 1,000 µM MeJA with 0.1% ethanol (vol/vol) as untreated control (CK). (C) DON production of PH-1 and *MRT1*^∆CT171^ mutant in LTB cultures with or without 1,000 µM MeJA at 7 dpi. (D) The morphology and growth inhibition rate of wild-type PH-1 and *MRT1*^S177A^, *MRT1*^S177D^, *MRT1*^T283A^, *MRT1*^T283D^ mutants at 3 dpi in PDA plates containing 1,000 µM MeJA with 0.1% ethanol (vol/vol) as untreated control (CK). Mean and standard deviation were estimated with data from three independent replicates with at least three cultures in each replicate. Asterisks and different letters indicate significant differences in comparison with CK or WT based on ANOVA (*P* < 0.05).

The *MRT1* gene encodes a 722-aa protein that harbors a GAL4 domain (29–66 aa) and a Fungal *Trans* domain (212–409 aa) (Fig. 4A). Mrt1 also has two predicted PKA phosphorylation sites, S177 and T283, that fit the PKA phosphorylation motif (S/R)(S/R)-X-(S/T) (Fig. 4A). Notably, the missense mutations (C34F and A371T) occurred within the GAL4 domain and the Fungal *Trans* domain, respectively (Fig. 4A). The nonsense mutations, W616* and W552*, led to the truncation of the C-terminal 107 or 171 amino acids of Mrt1 (Fig. 4A). The W552* mutation in mutant 52S resulted in the longest truncation (CT171, residues 552–722). To verify its impact on MeJA tolerance, we generated the *MRT1*^∆CT171^ gene replacement construct (Fig. 4A) and transformed it into the *mrt1* mutant. Compared to PH-1, the resulting *MRT1*^∆CT171^ mutant (Table 1) displayed enhanced resistance to MeJA in colony growth (Fig. 4B). These results confirmed that truncation of the C-terminal tail of Mrt1 confers MeJA tolerance. When assayed for DON production, the *MRT1*^∆CT171^ mutant was still sensitive to MeJA treatment (Fig. 4C). These data indicate that the truncation of CT171 only affects hyphal growth but not DON production in response to MeJA.

To determine the role of the potential PKA phosphorylation sites S177 and T283 in Mrt1, we introduced S177A, S177D, T283A, and T283D mutations by overlapping PCR, respectively (Fig. 4A). The resulting constructs were confirmed by sequencing analysis and subsequently transformed into *mrt1* mutant to obtain *MRT1*^S177A^, *MRT1*^S177D^, *MRT1*^T283A^, and *MRT1*^T283D^ transformants (Table 1). After 3 days of growth on PDA plates, all four transformants showed similar vegetative growth to the wild-type PH-1 (Fig. 4D). Notably, when exposed to 1,000 µM MeJA, only the *MRT1*^T283D^ transformant exhibited increased resistance to MeJA in fungal growth (Fig. 4D). These results indicate that the T283D mutation enhances tolerance against MeJA, suggesting that T283 may be the PKA phosphorylation site.

### The R178H mutation in *FgGPA1* increases tolerance to MeJA in fungal growth and DON production

**Fig 5:**
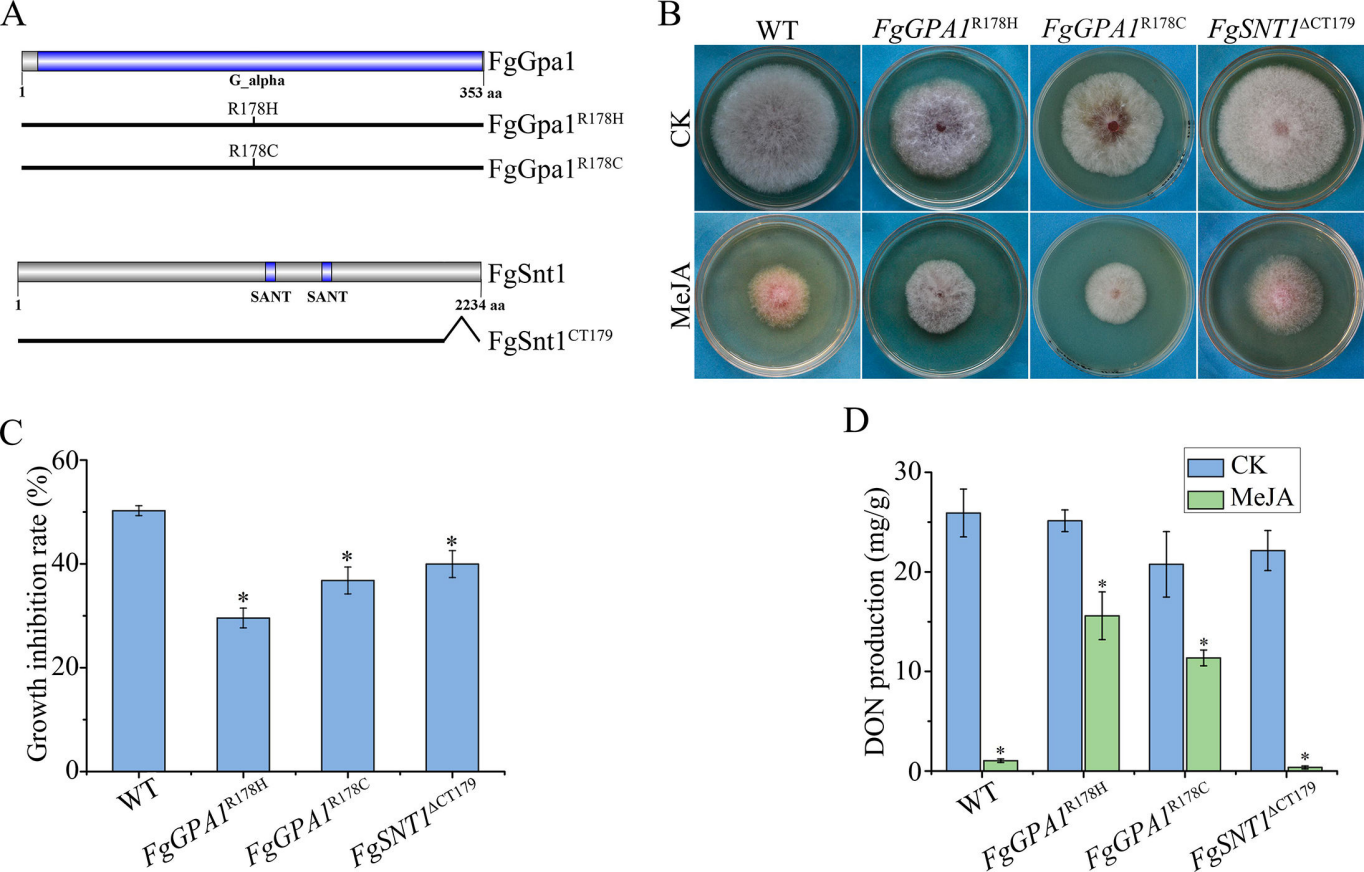
Mutations of *FgGPA1*^R178H^ and *FgSNT1*^∆CT179^ increase resistance to MeJA. (A) Schematic diagram of different mutant alleles of FgGpa1 and FgSnt1. (B) The morphology of wild-type (WT) PH-1 and *FgGPA1*^R178H^, *FgGPA1*^R178C^, and *FgSNT1*^∆CT179^ mutants in PDA plates containing 1,000 µM MeJA with 0.1% ethanol (vol/vol) as the untreated control (CK) at 3 dpi. (C) The growth of the same set of strains on PDA plates with or without MeJA was measured to estimate the growth inhibition rate. (D) DON production of PH-1 and *FgGPA1*^R178H^, *FgGPA1*^R178C^, and *FgSNT1*^∆CT179^ mutants in LTB cultures with or without 1,000 µM MeJA. Asterisks indicate significant differences in comparison with CK or WT based on ANOVA (*P* < 0.05).

Among the MeJA-tolerant spontaneous mutants, mutant 610S had a missense mutation R178H in the *FgGPA1* gene that encodes a G-alpha subunit (46). Therefore, it seems that the *FgGPA1*^R178H^ mutation confers MeJA resistance to *F. graminearum*. To confirm the effect of this mutation, we created the *FgGPA1*^R178H^ transformant (Table 1) by generating *FgGPA1*^R178H^ construct and transforming it into *Fggpa1* mutant (Fig. 5A). Similar to mutant 610S, the *FgGPA1*^R178H^ transformant showed increased tolerance against MeJA in colony growth (Fig. 5B and C). Because DON production was significantly reduced by MeJA, we assayed the effect of R178H mutation on DON production in the presence of 1,000 µM MeJA. In comparison to the wild type, the DON production was increased in *FgGPA1*^R178H^ transformant (Fig. 5D).

R178 in FgGpa1 is equivalent to R178 in the Gα subunit FadA of *Aspergillus nidulans* and Gna-1 of *Neurospora crassa*. The R178C mutation reduces the intrinsic GTPase activity of Gα, leading to a constitutive activation of Gna-1/FadA and higher intracellular cAMP (47, 48). Therefore, we also generated the *FgGPA1*^R178C^ mutant (Fig. 5A). Similar to R178H mutation, the R178C mutation alleviated the inhibitory effects of MeJA on fungal growth (Fig. 5B and C) and DON production (Fig. 5D).

### Truncation of the C-terminal tail in *FgSNT1* also increases tolerance to MeJA

Mutant 421S harbored a nonsense mutation Q2056* in *FgSNT1*, leading to the truncation of C-terminal 179 amino acids (CT179, residues 2,056–2,234). To confirm the effect of this mutation, we generated the *FgSNT1*^∆CT179^ transformant (Table 1) (Fig. 5A) and found that it had increased tolerance against MeJA, similar to the mutant 421S (Fig. 5B and C). However, the *FgSNT1*^∆CT179^ mutant was still significantly reduced in DON production when treated with 1,000 µM MeJA (Fig. 5D). These results indicate that truncation of CT179 increases tolerance to MeJA in colony growth but has no effect on the inhibitory effect of MeJA on DON biosynthesis.

### Treatments with MeJA are inhibitory to the cAMP-PKA signaling pathway

**Fig 6:**
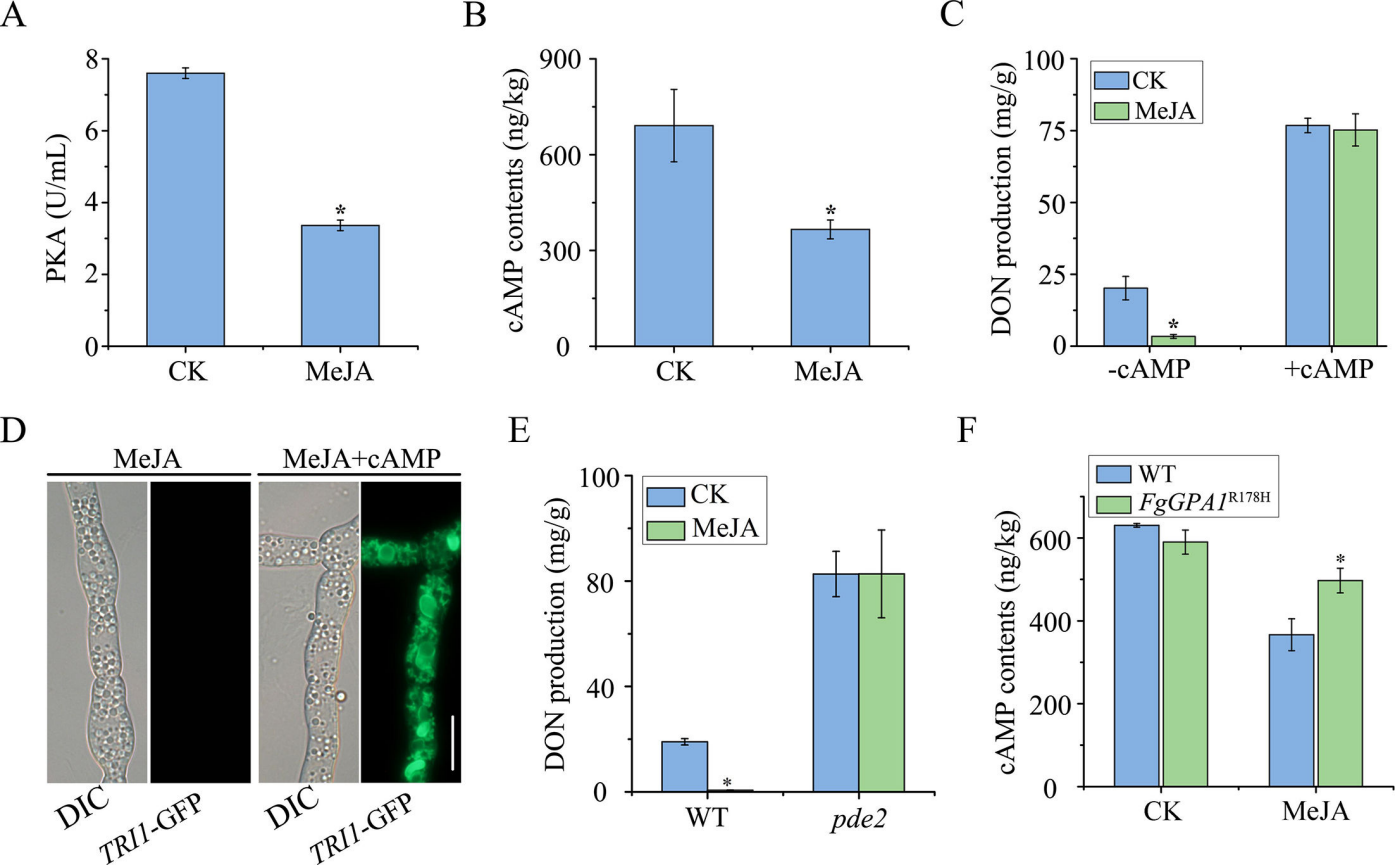
MeJA reduces the cellular PKA activity and cAMP level in hyphae. (A) PKA activity was assayed with proteins isolated from hyphae of the PH-1 supplemented with 0.1% ethanol (CK) or 1,000 µM MeJA in LTB cultures at 3 dpi. (B) The intracellular cAMP level of wild-type PH-1 treated with 1,000 µM MeJA was detected by UHPLC-MS/MS. (C) The PH-1 was incubated in LTB supplemented with 0.1% ethanol (CK) or 1,000 µM MeJA, both in the presence and absence of 4 mM cAMP, and then assayed for DON production at 7 dpi. Asterisk indicates significant differences with CK based on ANOVA followed by Tukey’s range tests (*P* < 0.05). (D) The inhibition of the expression of Tri1-GFP and toxisome formation by MeJA was restored by the addition of 4 mM cAMP. Bar = 10 µm. (E) DON production of PH-1 and *pde2* mutant in 7-day-old LTB cultures supplemented with 0.1% ethanol (CK) or 1,000 µM MeJA. (F) The intracellular cAMP level of wild-type PH-1 and *FgGPA1*^R178H^ mutant treated with 1,000 µM MeJA was assayed by UHPLC-MS/MS. Mean and standard deviation of the PKA activity and cAMP contents were estimated with data from three independent replicates. Asterisks indicate significant differences based on ANOVA (*P* < 0.05) compared to CK or WT.

Because *FgSNT1* and *FgGPA1* are related to the cAMP-PKA signaling pathway (49–51), and Mrt1 has two putative PKA-phosphorylation sites, we assayed the PKA activity in PH-1 treated with or without MeJA. When total proteins isolated from LTB cultures were assayed with the PKA (protein kinase A) Colorimetric Activity Kit (ThermoFisher, USA), the PKA activity in MeJA-treated hyphae was more than twofold lower than that in untreated control (Fig. 6A). We then assayed the intracellular cAMP level of PH-1 in LTB cultures. Treatments with MeJA reduced the intracellular cAMP level by approximately 41.1% in comparison to untreated control (Fig. 6B). These results indicated that MeJA treatment reduces the activation of the cAMP-PKA signaling pathway in *F. graminearum*.

### Activation of the cAMP-PKA pathway alleviates the inhibition of DON production caused by MeJA

Because the intracellular cAMP level was reduced in hyphae treated with MeJA, we investigated the impact of exogenous cAMP on DON production. Addition of 4 mM cAMP significantly enhanced DON production in LTB cultures treated with or without 1,000 µM MeJA (Fig. 6C). Furthermore, numerous toxisomes labeled with Tri1-GFP were observed in 3-day-old LTB cultures treated with both exogenous cAMP and MeJA (Fig. 6D). These results indicate that exogenous cAMP can suppress the inhibitory effect of MeJA on DON production.

In *F. graminearum*, Pde2 is the major cAMP phosphodiesterase, and *pde2* deletion mutant is increased in intracellular cAMP level and PKA activity (17). When treated with 1,000 µM MeJA, the *pde2* mutant produced as much DON as untreated control, unlike the wild type (Fig. 6E). The tolerance of the *pde2* mutant against MeJA confirms that the inhibitory effect of MeJA on DON production is dependent on the cAMP-PKA signaling pathway.

Given that the *FgGPA1*^R178H^ mutant was significantly increased in DON production when treated with MeJA, we examined the impact of the R178H mutation on cAMP-PKA pathway. When treated with MeJA, the *FgGPA1*^R178H^ transformant had a higher intracellular cAMP level than the wild-type strain PH-1 (Fig. 6F). Therefore, activation of the cAMP-PKA signaling pathway alleviates MeJA-induced inhibition of DON production.

### MeJA has no effect on the phosphorylation of MAP kinases

**Fig 7:**
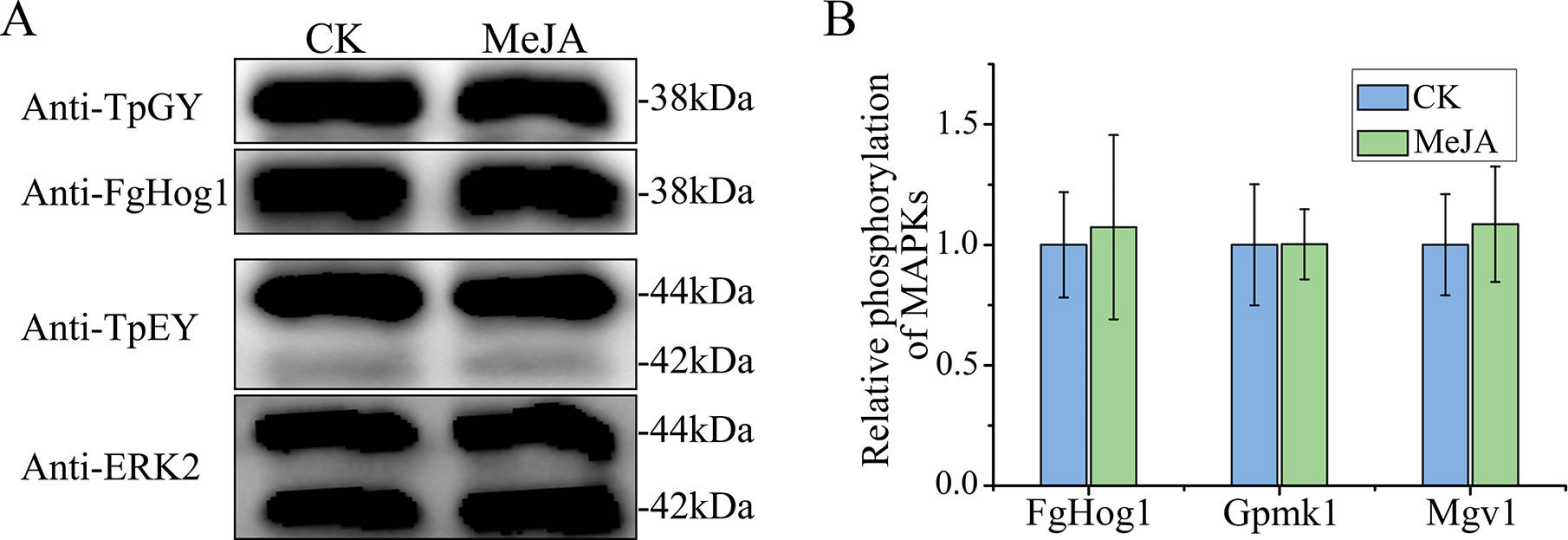
The effects of MeJA on the phosphorylation of the three MAP kinases FgHog1, Mgv1, and Gpmk1 in *F. graminearum*. (A) Total proteins isolated from hyphae of PH-1 in three-day-old LTB cultures treated with 0.1% ethanol (CK) or 1,000 µM MeJA for 30 min were detected with the anti-TpGY phosphorylation, anti-FgHog1, anti-TpEY phosphorylation, and anti-ERK2 antibodies. Molecular weights of FgHog1, Mgv1, and Gpmk1 are 38, 44, and 42 kDa, respectively. (B) The densities of the FgHog1, Gpmk1, and Mgv1 bands were analyzed with Image J Software to estimate changes in their phosphorylation levels. For each MAP kinase, its relative phosphorylation level in hyphae treated with 0.1% ethanol (CK) was set to 1.

In *F. graminearum*, all three MAP kinases, Gpmk1, Mgv1, and FgHog1, also play critical roles in regulating DON biosynthesis (30, 31). Thus, we assayed the effect of MeJA on the activation of these three MAP kinases. Western blots of proteins isolated from mycelia of PH-1 treated with 1,000 µM MeJA for 30 min were detected with the anti-FgHog1 and anti-TpGY antibodies. In PH-1 treated with MeJA, the phosphorylation level of FgHog1 remained unaltered in comparison to untreated control (Fig. 7A and B). Similarly, treatments with MeJA had no effect on the activation of Gpmk1 and Mgv1, two MAP kinases possessing the TEY phosphorylation site (Fig. 7A and B). These results indicate that MeJA has no obvious effect on these three conserved MAPK signaling pathways in *F. graminearum*.

### MeJA exhibits broad-spectrum antifungal activity against *Fusarium* species

**Fig 8:**
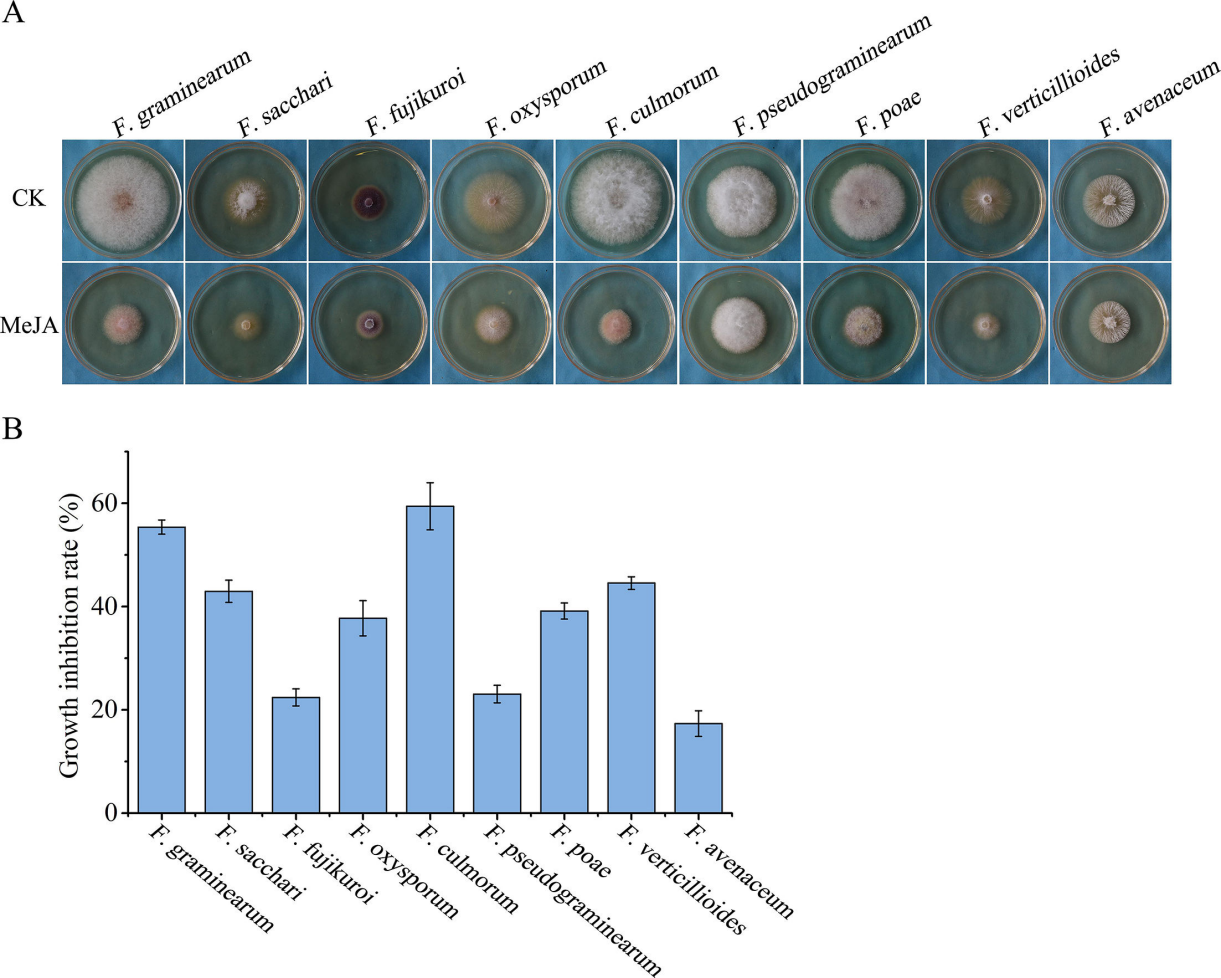
Broad-spectrum inhibitory effects of MeJA on multiple *Fusarium* species. (A) MeJA inhibited hyphal growth of eight additional *Fusarium* species. Colony morphology of labeled strains grown on PDA plates supplemented with 1,000 µM MeJA for 3 days. (B) The growth inhibition rates of indicated *Fusarium* strains treated with 1,000 µM MeJA on PDA plates. Mean and standard deviation were estimated with data from three independent replicates.

Because the cAMP-PKA signaling pathway is a target of MeJA and this pathway is conserved among *Fusarium* species, we evaluated the inhibitory effects of MeJA on the hyphal growth of eight additional *Fusarium* species, including *Fusarium sacchari*, *Fusarium fujikuroi*, *Fusarium oxysporum*, *Fusarium culmorum*, *Fusarium pseudograminearum*, *Fusarium poae*, *Fusarium verticillioides*, and *Fusarium avenaceum*. These *Fusarium* stains were cultured on PDA plates supplemented with 1,000 µM MeJA for 3 days. The results showed varying growth inhibition rates, ranging from 17.3% to 59.4%, with *F. culmorum* exhibiting the highest sensitivity (Fig. 8). These data indicate that MeJA effectively suppresses the hyphal growth in a wide range of *Fusarium* species, supporting its potential as a broad-spectrum antifungal agent.

## DISCUSSION

The wheat scab fungus *F. graminearum* causes huge yield losses and high food safety risks. However, the widespread application of synthesized chemicals causes environmental problems and health risks due to their residual toxicity. Thus, it is extremely urgent to develop efficient, safe, and eco-friendly agents or strategies to control *F. graminearum* growth and subsequent mycotoxin contamination.

MeJA is an important plant hormone regulating plant resistance to biotic and abiotic stresses (32, 33). MeJA has been extensively studied for preventing fungal diseases (52–55). As a plant-sourced and eco-friendly chemical, MeJA is widely applied to control post-harvest diseases, such as those caused by *Aspergillus flavus* in pistachios (56), *Botrytis cinerea* in strawberry (56), *Alternaria alternata* in cherry tomato (57), and *Colletotrichum acutatum* in loquat fruit (58). In this study, we evaluated the antifungal activity of MeJA to *F. graminearum* in *vitro* and found that MeJA could directly inhibit the mycelial growth, conidiation, and DON production in a dosage-dependent manner. This result further confirmed the previous findings that MeJA has an inhibitory effect on fungal pathogens, including *A. flavus* (59), *B. cinerea* (60), *Penicillium expansum* (61), and *C. acutatum* (58). However, in *Magnaporthe oryzae*, MeJA displays no obvious effect on hyphal growth (55). In *A. flavus*, MeJA at concentrations ranging from 10^−8^ to 10^−3^ M or higher can inhibit aflatoxin production (56, 59). However, in *Aspergillus parasiticus*, MeJA stimulates aflatoxin production at concentrations of 10^−6^ and 10^−4^ M (62, 63). It seems that the fungal inhibition by MeJA is species dependent. However, the underlying molecular mechanism is still unclear.

In some fungi, MeJA treatment triggers intracellular accumulation of ROS or disrupts membrane integrity (43, 59, 60), but these effects were not observed in *F. graminearum*, suggesting that MeJA may have other targets in *F. graminearum.* To uncover the underlying molecular mechanisms, chemical-genomic profiling, a powerful method to identify drug targets (64), was performed in this study. A total of 31 MeJA-resistant mutants were obtained from PDA plates containing MeJA. Mutated genes, including *MRT1*, *FgGPA1*, and *FgSNT1,* were identified in MeJA-resistant mutants by whole-genome sequencing analyses. Subsequently, we verified that mutations of *MRT1*^∆CT171^, *FgGPA1*^R178H^, and *FgSNT1*^∆CT179^ confer MeJA resistance in hyphal growth; however, only *FgGPA1*^R178H^ could increase DON production in the presence of MeJA. It is likely that these three mutations enhance resistance to MeJA by different mechanisms.

The Gpa1 is a Gα subunit of a heterotrimer (Gαβγ) that functions upstream of the cAMP-PKA signaling pathway in fungi (47, 48, 65). The *FgSNT1* gene encodes a component of Set3C HDAC complex, which is phosphorylated by the PKA catalytic subunit Cpk1 in *F. graminearum* (51). The Mrt1 may also be a PKA substrate since it contains two putative PKA phosphorylation sites (S177 and T283). We hypothesized that MeJA may target cAMP-PKA signaling pathway in a direct or indirect manner in *F. graminearum*. The cAMP-PKA pathway regulates DON biosynthesis through promoting *TRI* genes’ expression and toxisome formation (66, 67). Accordingly, we showed that MeJA treatments significantly inhibited the expression of *TRI* genes (*TRI1*, *TRI5*, and *TRI12*) and toxisome formation. Our results further demonstrated that MeJA treatment significantly reduced both intracellular cAMP level and PKA activity, which may be responsible for its antifungal activity. Furthermore, exogenous cAMP could reverse the inhibitory effects of MeJA on DON production. In *F. graminearum*, all three MAPKs, including Mgv1, Gpmk1, and FgHog1, are required for fungal growth and DON production (30, 68). However, our study showed that the MeJA treatment had no effects on MAPK signaling pathways. Therefore, cAMP-PKA signaling pathway may be the major target of MeJA in *F. graminearum*.

In this study, we demonstrated that both *FgGPA1*^R178H^ and *FgGPA1*^R178C^ mutations confer resistance to MeJA. In the Gα subunit FadA of *Aspergillus nidulans* or Gna-1 of *N. crassa*, the R178C mutation reduces the intrinsic GTPase activity of Gα subunit, causing higher cAMP levels and PKA pathway activation (47, 48). Thus, like R178C mutation, the *FgGPA1*^R178H^ mutation may also activate the cAMP-PKA pathway. Consistent with this, the R178H mutation of *FgGPA1* increases the intracellular cAMP level under MeJA treatments. Furthermore, the deletion of *PDE2* gene, the major cAMP phosphodiesterase, also confers MeJA resistance in DON production, further supporting the involvement of the cAMP-PKA pathway in the antifungal activities of MeJA. We speculated that the MeJA may target the upstream components of this pathway, including GPCR, Gαβγ heterotrimer, and regulators of G-protein signaling (RGS) proteins. Nevertheless, it is also possible that MeJA may act on the plasma membrane to disturb the endocytosis that also modulates the cAMP-PKA signaling pathway (69). In future studies, it will be important to further identify the exact targets of MeJA to better understand the underlying mechanisms.

We also noticed that *MRT1*^∆CT171^ and *FgSNT1*^∆CT179^ alleles only enhanced resistance to MeJA in vegetative growth but not in DON production. The Mrt1 is orthologous to the transcription factors MGG_04843 in *M. oryzae*, Ads-1 in *N. crassa,* and Mdu2 in *Aspergillus fumigatus,* all of which are involved in antifungal drug resistance (70–73). Moreover, the FgSnt1, a component of Set3C HDAC complex, regulates the gene expression through H4 acetylation (51). It is likely that the *MRT1*^∆CT171^ and *FgSNT1*^∆CT179^ mutations may enhance the efflux or degradation of MeJA by changing transcriptional expression. Since the FgSnt1 is phosphorylated at S433 by Cpk1 (51) and Mrt1 has two putative PKA-phosphorylation sites, it seems that Mrt1 functions downstream of the cAMP-PKA pathway as well as FgSnt1. Consistent with this, mutating the putative PKA phosphorylation site (T283D) of Mrt1 to mimic a phosphorylated state could alleviate the MeJA-mediated inhibition in vegetative growth. Moreover, deleting the C-terminal 98 aa or introducing a phospho-mimicking mutation (S433D) at the PKA phosphorylation site in FgSnt1 can reprogram transcription to rescue the defects of *pkr* mutant (51). Thus, the C-terminal 179 aa deletion of FgSnt1 may have a similar effect on activating the transcription of downstream genes to MeJA. Overall, our results showed that treatment with MeJA is inhibitory to *F. graminearum* on growth, conidiation, and DON production, which may be directly related to its inhibitory effect on the cAMP-PKA signaling pathway.

This study highlights the inhibitory effects of MeJA on both *Fusarium* growth and DON synthesis, establishing a promising foundation for its application in food safety and agricultural practices. Based on these findings, future research could focus on evaluating the effectiveness of MeJA in mitigating DON toxin contamination in stored grains and silage, where *Fusarium* poses substantial risks. Furthermore, efforts to chemically modify MeJA to develop more effective and targeted fungicides could significantly enhance its applicability in managing *Fusarium*-related diseases. These advancements would not only broaden the practical applications of MeJA but also contribute to enhancing food safety and minimizing the detrimental impacts of fungal toxin contamination.

## MATERIALS AND METHODS

### Strains and cultural conditions

The wild-type strain PH-1 (41) and transformants generated in this study were routinely cultured on potato dextrose agar at 25°C (74). To determine the inhibitory effects of jasmonates on fungal growth, MeJA and JA (Aladdin, Shanghai, China) were added to PDA to final concentrations of 30, 500, 1,000, and 4,000 µM. JA and MeJA were dissolved in 100% ethanol as the stock solutions and assayed with 0.1% ethanol (vol/vol) as the untreated control (CK). Hyphae in 24-h YEPD cultures with different concentrations of MeJA were collected to lyophilize in a Heto Power dryer PL3000 (Thermo, Waltham, MA, USA) and measured for dry weights as described (75). Conidiation was assayed in 5-day-old CMC cultures as described (76). Protoplast preparation and transformation were performed as described in a previous study (77, 78).

### Assays for the effects of MeJA on DON biosynthesis and *TRI* gene expression

To assay the effect of MeJA on DON production, MeJA was added to the final concentrations of 30, 100, 200, 500, and 1,000 µM to 2 mL LTB cultures with 1 × 10^4^ conidia/mL as described (42). After incubation for 7 days at 25°C in the dark, DON toxin was extracted from LTB cultures and analyzed with a GCMS-QP2010 (Shimadzu, Kyoto, Japan) as described (42). A similar approach was performed to assay DON production in the inoculated wheat spike at 14 dpi. The swollen hyphal compartments and toxisome formation were observed in hyphae from 3-day-old LTB cultures (17). To detect the *TRI* gene expression, hyphae from 3-day-old LTB cultures with or without 1,000 µM MeJA were used to isolate RNA with the Eastep Super Total RNA Extraction Kit (Promega, Madison, WI, USA), following the manufacturer’s instructions. The cDNA was synthesized with the FastKing RT Kit (Tiangen, Beijing, China). The Taq SYBRGreen qPCR Premix (Yugong Biotech, Lianyungang, China) was used to assay the expression of *TRI* genes (*TRI1*, *TRI5*, and *TRI12*) with the CFX96 Real-Time System (Bio-Rad, Hercules, CA, USA) (17). The actin gene was amplified with the primers actin-RT-F and actin-RT-R as the endogenous reference (79). Three independent biological replicates were performed for each sample with three technical replicates each. The 2^–ΔΔCT^ method was performed to calculate the relative gene expression (80).

### Assays for intracellular ROS level

To assay the intracellular level of ROS in hyphae, the reactive oxygen species assay kit (Beyotime, Haimen, China) was used following the instructions provided by the manufacturer. In brief, the fresh conidia of wild-type PH-1 were cultured in YEPD medium for 8 h and subsequently subjected to treatment with either 1,000 µM MeJA or 0.1% ethanol for 30 min (CK). After treatment, the hyphae were collected by filtration with a miracloth (Calbiochem, San Diego, CA, USA) and resuspended in a solution containing 10 µM of DCFH-DA. After incubation at 37°C for 30 min, the hyphae were observed under an Olympus BX53 Fluorescence Microscope equipped with a DP80 digital camera (Olympus, Tokyo, Japan). The fluorescence intensity of the intracellular ROS was determined using the Victor Nivo Microplate Reader (PerkinElmer, Waltham, MA, USA) with the excitation wavelength set at 480 nm and the emission wavelength at 530 nm.

### Assays for cell viability

The PI stain was used to determine the effect of MeJA on cell viability of the PH-1 strain. Fresh conidia of PH-1 were transferred to 20 mL YPED with or without 1,000 and 4,000 µM MeJA and cultured on a rotary shaker at 175 rpm and 25°C for 12 h. The hyphae harvested by filtration were washed with PBS buffer (pH 7.4) three times and stained with 200 µL of 5 µg/mL PI for 30 min in the dark as described (81). After incubation, the hyphae were rinsed with PBS buffer and observed under an Olympus BX53 Fluorescence Microscope (Olympus, Tokyo, Japan). The hyphal sample was treated at 100°C for 3 min and stained with PI as the positive control.

### Quantification of PKA activity and intracellular cAMP level

To assess the effects of MeJA on PKA activities or intracellular cAMP levels, 3-day-old LTB cultures were treated with or without 1,000 µM MeJA for 30 min and filtered through miracloth (Sigma, St. Louis, MO, USA) to harvest hyphae samples. For detecting the PKA activities, the harvested hyphae were washed with sterile distilled water and used to isolate total protein as described (82). The PKA activities were assayed with the PKA (Protein Kinase A) Colorimetric Activity Kit (ThermoFisher, Waltham, MA, USA) following the manufacturer’s instructions. For detecting the intracellular cAMP level, the harvested hyphae were frozen in liquid nitrogen and lyophilized for 24 h with Heto Powerdryer PL3000 (ThermoFisher, Waltham, MA, USA). After grinding in liquid nitrogen, 40 mg of hyphal powder was resuspended in 800 µL of ice-cold 6% trichloroethanoic acid and incubated on ice for 10 min. After centrifugation at 4,000 rpm for 10 min at 4°C, the supernatant was collected and washed four times with five volumes of water-saturated diethyl ether. The remaining aqueous phase was filtered through a 0.22 µm hydrophilic membrane (Shimadzu, Kyoto, Japan). Finally, 5 µL of the filtrate was injected into a UHPLC coupled with a QTRAP 5500 (AB Sciex, Ontario, Canada) with a C18 column (3.0 × 100 mm, 3 µm). The gradient elution was set at 90% A and 10% B initially, maintained for 0.5 min, increased to 70% B at 4.0 min, maintained for 1 min, and returned to 10% B at 5.5 min. The flow rate was 0.4 mL/min, and the injection volume was 5 µL. Eluates were detected using a triple quad 5500^+^ mass spectrometer (AB Sciex, Ontario, Canada) in the positive electrospray ionisation mode by MRM. The settings for the ion source were as follows: entrance potential: 10 V, collisionally activated dissociation gas: 6 psi, curtain gas: 35 psi, nebulizer gas (gas 1): 50 psi, turbo gas (gas 2): 55 psi, ion spray voltage: 5,500 V, and source temperature: 500°C. The analysis was carried out with an operating pressure of 3.9 × 10^−5^ Torr. Qualitative and quantitative analyses with precursor ion 330*m/z*, product ion 136 and 119 *m/z.* The Analyst 1.7.0 (AB Sciex, Ontario, Canada) was used for data acquisition and processing.

### Generation of the *mrt1*, *Fggpa1*, and *FgSNT1^∆^*^CT179^ mutants

To generate the ∆*mrt1* mutants, the split-marker approach was performed as previously described (83). The 0.75-kb upstream and 0.72-kb downstream flanking fragments were amplified with primer pairs MRT1-1F/2R and MRT1-3F/4R, respectively. Two hygromycin phosphotransferase (*hph*) fragments (H1 and H2) were amplified with primer pairs HYG-F/HY-R and YG-F/HT-R from PHH plasmid, respectively. Subsequently, the upstream and downstream fragments of *MRT1* were fused to the H1 and H2 fragments by overlapping PCR as described (84) and were transformed into the wild-type strain PH-1. Hygromycin-resistant transformants were verified for *MRT1* deletion by PCR with primer pairs H850/H852, MRT1-5F/6R, MRT1-7F/H856R, and HT855F/MRT1-8R. The same approach was performed to generate the ∆*Fggpa1* and *FgSNT1^∆^*^CT179^ mutants. All the primers are listed in Table S2.

### Generation of the *MRT1^∆^*^CT171^, *FgGPA1*^R178H^, *FgGPA1*^R178C^, *MRT1*^S177A^, *MRT1*^S177D^, *MRT1*^T283A^, and *MRT1*^T283D^ transformants

To generate the *MRT1^∆^*^CT171^ construct in which the C-terminal 171 aa was deleted, PCR product amplified with primer pair MRT1-CT171-F/R was cloned into the *Kpn* I/*Hind* III double-digested pKNTG vector with the geneticin-resistance marker using the Hieff Clone Plus One Step Cloning Kit (Yeasen Biotechnology, Shanghai, China) to gain the *MRT1^∆^*^CT171^ construct. The resulting *MRT1^∆^*^CT171^ construct was transformed into the *mrt1* mutant to obtain the *MRT1^∆^*^CT171^ transformants. The R178H mutation in *FgGPA1* was introduced by overlapping PCR as described (85) with primer pairs GPA1-R178-1F/GPA1-R178H-2R and GPA1-R178H-3F/GPA1-R178-4R (Table S2). The resulting PCR product was cloned into the *Kpn* I/*Hind* III double-digested pKNTG vector (86). The resulting *FgGPA1*^R178H^ construct was transformed into the *Fggpa1* mutant to obtain the *FgGPA1*^R178H^ transformants. The same approach was performed to generate the *FgGPA1*^R178C^, *MRT1*^S177A^, *MRT1*^S177D^, *MRT1*^T283A^, and *MRT1*^T283D^ transformants. All the primers used are listed in Table S2.

### MAPK phosphorylation assays

The MAPK phosphorylation level in hyphae affected by MeJA were detected as described (42). Hyphae of PH-1 were harvested from 3-day-old LTB cultures with 1,000 µM MeJA or 0.1% ethanol (vol/vol) treatment for 30 min. Total proteins were isolated with protein lysis buffer containing protease inhibitor cocktail, phosphatase inhibitor cocktail 2 and phosphatase inhibitor cocktail 3 (all from Sigma-Aldrich, St. Louis, MO, USA) and separated on 10% PAGE gels as described (87). TEY phosphorylation of Mgv1 and Gpmk1 was detected with the PhosphoPlus p44/42 MAPK (Erk1/2) (Thr202/Tyr204) Antibody Kit (Cell Signaling Technology, Danvers, MA, USA) following the manufacturer’s instructions. The TGY phosphorylation of FgHog1 was detected with the PhophoPlus p44/42 and p38 MAP kinase antibody kits (Cell Signaling Technology, Danvers, MA, USA) (87). The expression level of Gpmk1, Mgv1, and FgHog1 was detected with the anti-Erk2 and anti-FgHog1 polyclonal antibodies. Band densities were analyzed with the Image Lab software. Each experiment was repeated three times independently.

## SUPPLEMENTAL MATERIAL

Supplemental material mbio.03151-24-s0001.pdf Supplemental figures and tables.

ASM does not own the copyrights to Supplemental Material that may be linked to, or accessed through, an article. The authors have granted ASM a non-exclusive, world-wide license to publish the Supplemental Material files. Please contact the corresponding author directly for reuse.
