# Supplementary material for: MeJA inhibits fungal growth and DON toxin production by interfering with the cAMP-PKA signaling pathway in the wheat scab fungus Fusarium graminearum
Source: mBio. 2025 Feb 4;16(3):e03151-24. doi: 10.1128/mbio.03151-24 (PMC11898702; doi:10.1128/mbio.03151-24)
Supplement: Supplemental material — Supplemental figures and tables. [file mbio.03151-24-s0001.pdf]

Supplemented Informations

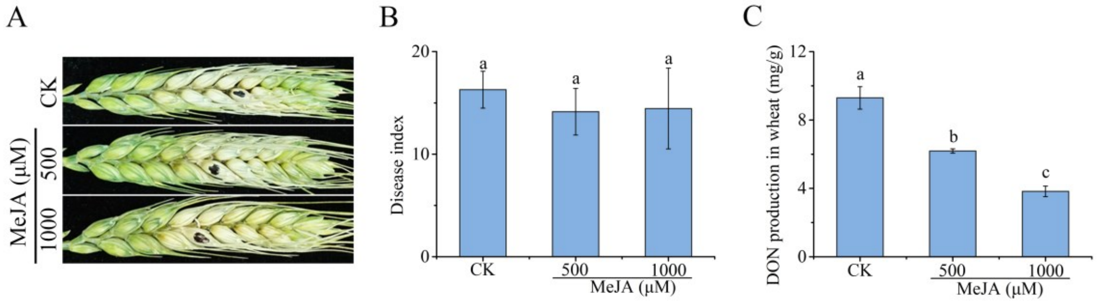

Fig. S1. Inhibitory effects of MeJA on the virulence of *F. graminearum* and DON production *in planta*.

A. Flowering wheat heads were drop-inoculated with conidium suspensions of PH-1 with the marked concentrations (500 and 1000 μM) of MeJA and photographed at 14 dpi. Black dots mark the inoculation site. **B.** Disease index was estimated by counting the number of diseased wheat kernels. At least 10 wheat heads were inoculated for each strain. **C.** DON production in infected wheat kernels was detected with GC-MS. Different letters indicate significant differences based on ANOVA analysis followed by Tukey's range tests ($p<0.05$).

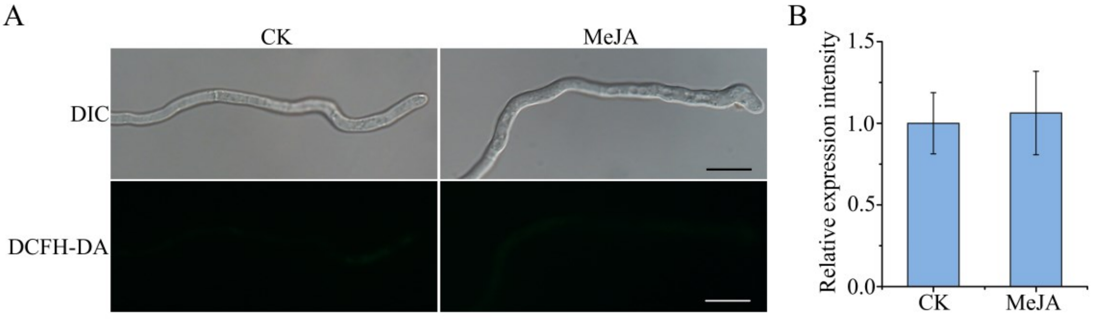

Fig. S2. The effect of MeJA treatment on ROS accumulation of *F. graminearum*.

A. PH-1 hyphae harvested from 8-h YEPD cultures were treated with 1000 μM MeJA or 0.1% ethanol (CK) for 30 min and stained with dichloro-dihydro-fluorescein diacetate (DCFH-DA) before being examined by DIC and fluorescence microscopy.

Bar=10 μm. **B.** Relative fluorescence intensity of ROS accumulation in hyphae was measured with a micro-plate reader. The relative level of ROS in cultures with 0.1% ethanol (CK) was set to 1. Mean and standard deviation of the fluorescence intensity were estimated with data from three independent replicates.

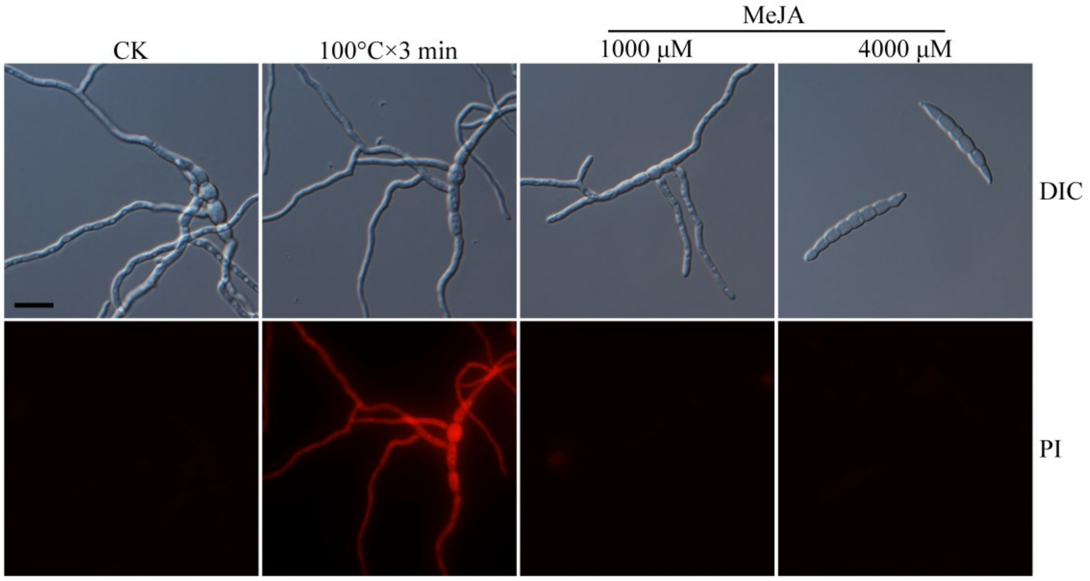

Fig. S3. The effect of MeJA treatment on cell viability of *F. graminearum*. Conidia of wild-type PH-1 (WT) were cultured in YEPD supplemented with or without 1000 and 4000 μM MeJA or 0.1% ethanol (CK) for 12 h and stained with 5 μg/ml propidium iodide (PI). The hyphal sample treated at 100°C for 3 min was stained with PI as the positive control. Bar=20 μm.

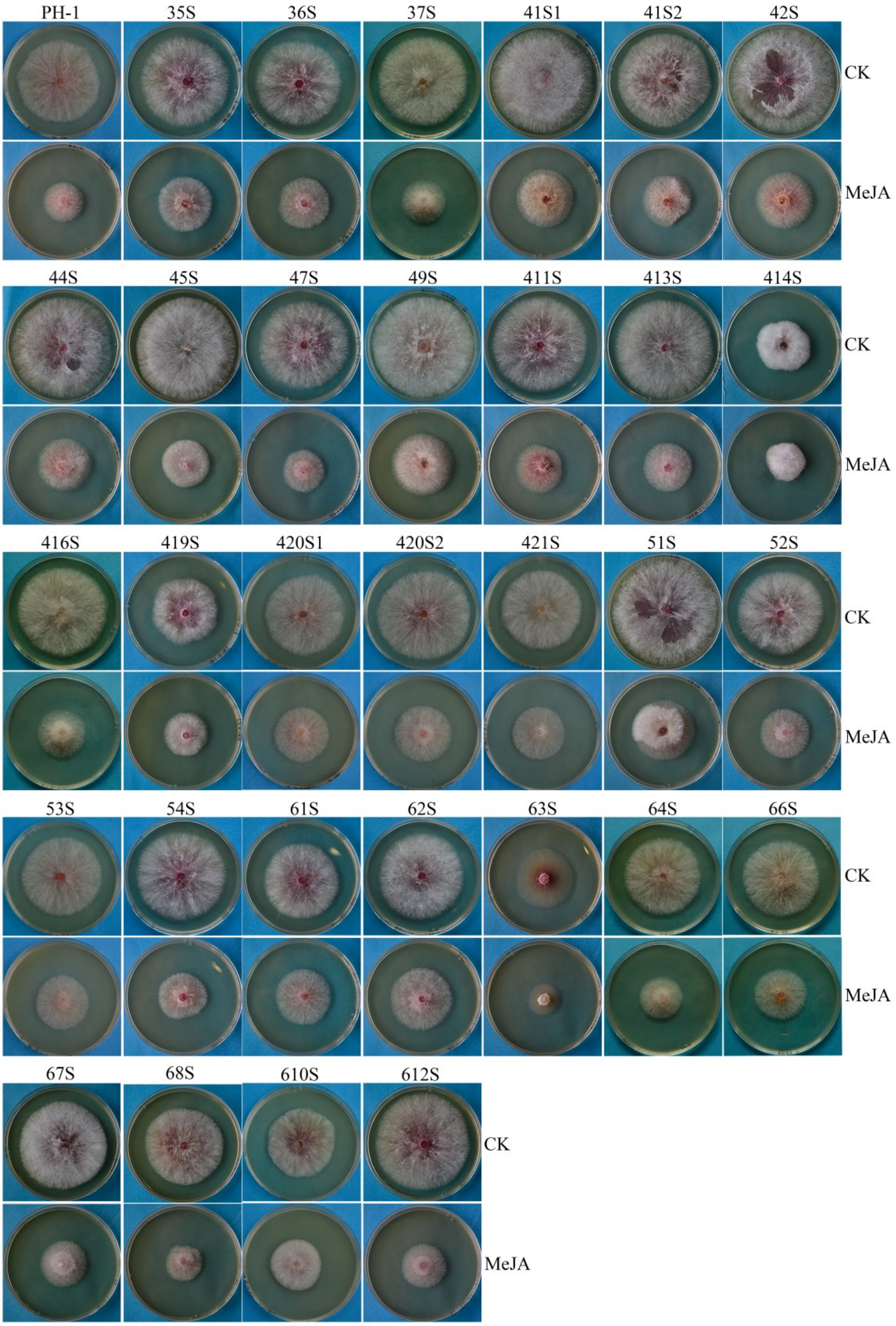

Fig. S4. The colony morphology and growth of all MeJA-resistant mutants.

A total of 31 MeJA-resistant mutants were re-examined in PDA supplemented with or without 1000 μM MeJA at 3 dpi in order to determine the growth inhibition rates.

Table S1. The colony growth and growth inhibition rate of all MeJA-resistant mutants

Strains	Growth rate (mm/d)		Growth inhibition rate (%)
	CK	MeJA	
PH-1	11.7±0.2	5.2±0.1	55.7±0.2
610S	8.2±0.3	5.8±0.5	25.5±2.9
66S	10.1±0.1	5.6±0.2	34.8±1.9
421S	10.1±0.1	6.3±1.0	31.8±3.5
62S	11.6±0.6	7.0±0.9	39.9±7.8
420S2	11.5±0.2	7.5±0.6	34.6±4.9
416S	11.2±0.3	7.1±0.4	37.1±3.7
53S	10.3±0.7	6.7±0.3	35.1±2.4
420S1	10.1±0.4	6.4±0.5	36.6±4.7
52S	10.9±0.2	6.2±0.2	39.7±1.9
35S	11.5±0.1	6.7±0.3	40.1±2.3
41S2	11.9±0.2	7.1±0.7	40.2±5.5
61S	10.1±0.4	5.8±1.4	41.9±14
36S	11.1±0.1	6.3±0.2	42.7±2.1
54S	11.3±0.2	6.4±0.4	43.1±3.5
419S	9.0±0.4	5.1±0.6	43.1±6.2
414S	12.4±0.2	7.0±0.8	43.2±6.6
64S	10.1±0.1	5.6±0.3	44.8±2.5
411S	10.8±1.4	6.1±0.2	43.8±1.5
42S	13.5±0.1	7.6±0.4	43.8±2.7
51S	13.3±0.2	7.3±0.6	44.4±3.1
49S	13.8±0.1	7.6±0.4	44.8±3.2
63S	7.3±0.7	4.1±0.1	44.0±1.5
47S	12.7±0.5	7.1±0.2	44.0±1.8
41S1	13.5±0.3	7.4±0.1	45.3±0.6
37S	11.3±0.4	5.8±0.3	48.5±2.5
413S	11.5±0.3	5.6±0.1	51.2±0.8
44S	12.8±0.3	6.6±0.8	48.6±6.1
67S	11.0±0.3	5.6±0.4	48.7±3.6
45S	12.8±0.2	6.3±0.4	50.5±3.1
68S	9.7±0.2	4.6±0.3	52.9±2.7
612S	12.3±0.3	5.7±0.1	53.5±0.5

Table S2. Primers used in this study

Primer name	Sequence (5'-3')
TRI1QRT-F	TCATCAAGCCCCTGCAGGAAGAG
TRI1QRT-R	CACCATTGAAGCAACTTGAATG
TRI5QRT-F	GATACAGAGGACGCCAAGAAG
TRI5QRT-R	CGAACGTTTGCCAGTTGTG
TRI12QRT-F	GCCAGAGCGATAACCAAAGT
TRI12QRT-R	GTCGCCCAAATCTATCCGTAAG
actin-RT-F	ATCCACGTCACCACTTTCAA
actin-RT-R	TGCTTGGAGATCCACATTTG
TRI1-GFP-F	AGGGAACAAAAGCTGGGTACCGCTATACTCGGCAGTCCTTTGC
TRI1-GFP-R	GAACAGCTCCTCGCCCTTGCTCACGGCGTCATTTGGGCTTGAGATAG
MRT1-1F	TCGATGCCCTGACTGTTGAG
MRT1-2R	TTGACCTCCACTAGCTCCAGCCAAGCCCCGATATGTTTCGCTCGGTC
MRT1-3F	CGTCCGCAATGTGTTATTAAGTCGACTGATGATGATGGCTTGCTGTGA
MRT1-4R	CCGATCTTCCACAGCACTCAAA
MRT1-5F	AAGACGTGCCAAACGACTCC
MRT1-6R	AAGCTCTGGACTTTTGTGGCA
MRT1-7F	GGTCCTTGGAATCAGCCAGTT
MRT1-8R	ATAACGCGAACCATGGCAGA
MRT1-CT171-F	ACGAGCTGTACAAGTAAGGATCCCTTTATGTTTGCATGTGACATCCATTT
MRT1-CT171-R	ATGATTTCAGTAACGTTAAGTCTAAAGAAAATTTCCCTGGCTAGCC
MRT1-F	AGGGAACAAAAGCTGGGTACCCCAATAATTAACAACTCTACTGATCAAAG
MRT1-R	GAACAGCTCCTCGCCCTTGCTCACCCAAGGCGACAATATTGAAGG
MRT1-S177A-R	CGTCTTCGTGTGTAACGGGTGCCGCCCGTGAAGGACATG
MRT1-S177A-F	GAAACATGTCCTTCACGGGCGGCACCCGTTACACACGAAGAC
MRT1-S177D-R	CGTCGTCTTCGTGTGTAACGGGGTCCGCCCGTGAAGGACATG
MRT1-S177D-F	GAAACATGTCCTTCACGGGCGGACCCCGTTACACACGAAGAC
MRT1-T283A-R	TTGAGTGCTGTTTTAGACCATCCGCGAACCGCCGAATGGCTT

MRT1-T283A-F	GAATGAAGCCATTCGGCGGTTCGCGGATGGTCTAAAAACAGCACT
MRT1-T283D-R	TTGTTGAGTGCTGTTTTTAGACCATCGTCGAACCGCCGAATGGCTT
MRT1-T283D-F	GAATGAAGCCATTCGGCGGTTCGACGATGGTCTAAAAACAGCACT
GPA1-1F	ACTTTTACCTTTTGTACCGCTCCC
GPA1-2R	<u>TTGACCTCCACTAGCTCCAGCCAAGCC</u>TGTGTCGTTGTCGATGTCGC
GPA1-3F	<u>CGTCCGCAATGTGTTATTAAGTCGAC</u>TGCTGTCACTAAAACACGAATACC
GPA1-4R	CATGGAGACAAAGTCACATGACTG
GPA1-5F	TGCGGAATGAGCACAGAGG
GPA1-6R	AAGACCACAGAGACGAAGGTTCTC
GPA1-7F	CATCTCGTCTCATTTTTCTTCTGC
GPA1-8R	ATGTTGTCTTGAACCGCCATC
GPA1-R178-1F	CATGGACGAGCTGTACAAGTAATGGGATCTTCGGCAAAGCTAG
GPA1-R178H-2R	GATACCTGTGGTCTTGACATGAGATCGGAGAACGTCCTG
GPA1-R178H-3F	CAGGACGTTCTCCGATCTCATGTCAAGACCACAGGTATC
GPA1-R178-4R	GATTTCAGTAACGTTAAGTGGATCCTTAGATAAGACCACAGAGACGAAGG
GPA1-R178C-2R	CAGGACGTTCTCCGATCTTGTGTCAAGACCACAGGTATC
GPA1-R178C-3F	GATACCTGTGGTCTTGACACAAGATCGGAGAACGTCCTG
SNT1-CT179-1F	CGAGATGACACCACAGCCT
SNT1-CT179-2R	<u>TTGACCTCCACTAGCTCCAGCCAAGCC</u>CTACATGCTGGGATGACCTTG
SNT1-CT179-3F	<u>CGTCCGCAATGTGTTATTAAGTCGAC</u>GTTCGGTGCAAGCAAACATG
SNT1-CT179-4R	GCTCTTGCATCAATGACTTCC
SNT1-CT179-5F	GCCATCGCAACCAAACAC
SNT1-CT179-6R	CCTCTCATCATGTCTCGAGG
SNT1-CT179-7F	CCATTGCAAGCACCTGTACAAC
SNT1-CT179-8R	GTTGCGAACTAGATACGAAACGG
HYG-F	GGCTTGGCTGGAGCTAGTGGAGGTCAA
HY-R	GTATTGACCGATTCCTTGCGGTCCGAA
YG-F	GATGTAGGAGGGCGTGGATATGTCCT
HT-R	GTCGACTTAATAACACATTGCGGACGT
H850	TTCCTCCCTTTATTTCAGATTCAA
H852	ATGTTGGCGACCTCGTATTGG
H855F	GTCGATGCGACGCAATCGT
H856R	GCTGATCTGACCAGTTGC

37
